# Low-priority items are held in visual working memory: Evidence from flexible allocation in a two-alternative forced-choice (2AFC) paradigm

**DOI:** 10.1167/jov.25.3.5

**Published:** 2025-03-07

**Authors:** Holly A. Lockhart, Stephen M. Emrich

**Affiliations:** 1 Department of Psychology, Brock University, St. Catharines, ON, Canada

**Keywords:** visual working memory, continuous recall, signal detection, resources

## Abstract

Visual working memory (VWM) is characterized as extremely capacity limited. This finding is supported by the dramatic decline in change detection performance beyond a small number of items, as well as the observation of flat error distributions in delayed-estimation tasks. However, continuous resource models predict that small amounts of memory resources can be distributed to items at the expense of memory resolution (resulting in low response precision). These low-resolution memory representations should have nearly flat error distributions that could appear indistinguishable from uniform guessing distributions. In the current study, memory resource allocation was manipulated by varying the probability of an item being probed at recall. Responses were intermixed between continuous response and two-alternative forced-choice (2AFC) trials to examine whether these low-probability items could produce above-chance performance, consistent with them being held in memory. For comparison with the distribution of continuous responses, the magnitude of the discrimination between the target and lure colors was manipulated. Accuracy on the 2AFC trials was sensitive to both discrimination difficulty and probe probability manipulations. Critically, above-chance performance was found in the lowest probe probability condition (10% probe probability, equivalent to an item load of 10), suggesting that this condition had low-resolution memory representations rather than no memory representations. These findings are consistent with the predictions of continuous resource models and applications of signal detection models of VWM.

## Introduction

Visual working memory (VWM) is the system for keeping visual information “in mind” for immediate use; however, it is severely capacity limited. The effect of increasing set size on performance—namely, to reduce accuracy, reduce precision, and increase reaction time—is essentially universal and is recognized as a benchmark property of VWM (Oberauer et al., 2018). However, the exact nature of this limitation remains debated, with several models attempting to explain these effects (Alvarez & Cavanagh, 2004; Bays, Catalao, & Husain, 2009; Oberauer & Lin, 2017; Schurgin, Wixted, & Brady, 2020; van den Berg & Ma, 2018; Zhang & Luck, 2008).

The predominant models explaining set size effects are discrete (or item-limit) models. Discrete resource models evolved out of the earliest work characterizing VWM capacity using change detection. In these studies, as set size increased, the ability to correctly assess whether an item in a memory array changed decreased, systematically at first and then dramatically beyond three or four items (Luck & Vogel, 1997). Conceptually, this suggests that there are two distinct categories of items: in-memory items (up to memory capacity) and not-in-memory items (beyond memory capacity). However, estimating capacity through change detection performance ignores “partial information” in memory in favor of a simplified all-or-none memory store (Pashler, 1988).

This conceptualization of memory as all-or-none is incongruent with continuous resource models. Continuous resource models do not propose a strict item-based capacity limit; instead, these models account for decreasing performance at higher memory loads by suggesting that items receive a smaller proportion of limited memory resources (Bays & Husain, 2008; Bays et al., 2009). Continuous resource models fit naturally within the context signal detection models, which theorize a system with both signal and normally distributed noise around that signal: Decreased accuracy with increasing set size reflects the relative increase in neural noise with higher memory loads (Bays, 2014; Bays, 2015; Luck & Vogel, 2013; Ma, Husain, & Bays, 2014; Wilken & Ma, 2004). At higher memory loads, the population of neurons that are encoding for one item may be competing with the neurons encoding for another item. If there is a limited number of neurons available to encode a memory array, this competition would result in fewer neurons being dedicated to any single memory item and thus less signal per item. Additionally, multiple concurrent memory representations may interfere with each other such that the *signal* for one representation is *noise* for the other (e.g., Oberauer & Lin, 2017). When examining performance at high memory loads it becomes difficult to distinguish the effects of decreasing resource allocation from relatively increasing neural noise.

One approach to separating the effects of item load from resource allocation is through attentional prioritization. That is, according to flexible resource models, a fully continuous resource should be able to be flexibly allocated according to one's goals, rather than being determined solely by the number of items one is attempting to remember (Emrich, Lockhart, & Al-Aidroos, 2017; Klyszejko, Rahmati, & Curtis, 2014). It has been shown that memory resources can be flexibly allocated in a manner that mimics item-based memory load increases (i.e., increased set size) by manipulating the likelihood that a given item will be tested (Dube, Emrich, & Al-Aidroos, 2017; Emrich et al., 2017). A fully flexible resource that tracts with attentional priority (e.g., probe probability) predicts that response precision should be in line with the proportion of resources allocated to each item as would be predicted by a load manipulation; for example, in a memory load of two items, each item should receive approximately 50% of memory resources. Emrich et al. (2017) demonstrated this effect in a delayed-estimation task across cue-validity conditions ranging from 9% to 100%, and similar effects have been observed using feature-based cues (Dube et al., 2017; Salahub, Lockhart, Dube, Al-Aidroos, & Emrich, 2019) and reward (Klyszejko, Rahmati, & Curtis, 2014), as well as in a memory-guided saccade task (Yoo, Klyszejko, Curtis, & Ma, 2018) and single- and dual-target visual search (Huynh Cong & Kerzel, 2022). Thus, these studies have demonstrated that memory resources can be flexibly allocated via attention, and individual items can receive a small proportion of resources, consistent with a continuous-resource model of VWM.

However, a weakness of continuous response paradigms is it is difficult to distinguish a very wide error distribution from a truly uniform distribution, the latter of which is indicative of random guesses. Often error distributions for very low-priority items are very wide and flat with only some central tendency (e.g., Emrich et al., 2017). Adam, Vogel, and Awh (2017) found similarly wide and flat distributions in a whole report task for the fourth to sixth responses and interpreted these distributions as uniform distributions reflecting guessing. Adam et al. (2017) calculated that it would take “over 900 million trials” (p. 94) to distinguish between low-precision memory and guessing in a six-item whole report. In contrast, alternative forced-choice tasks are often used in investigations of long-term memory specifically because they can reveal evidence of “weak” memory through more simple binary judgments (Brady, Robinson, Williams, & Wixted, 2023; Schurgin, 2018). Results from these binary judgments could provide evidence of low-resolution memory representations by using principles of signal detection theory as understood through continuous resource models.

Although most studies examining the possible continuous nature of VWM resources have tended to use delayed-estimation paradigms, some have used change detection (Keshvari, van den Berg, & Ma, 2013; Pearson, Raskevicius, Bays, Pertzov, & Husain, 2014), or change localization (van den Berg, Shin, Chou, George, & Ma, 2012). In studies of change detection and change localization, researchers use a psychometric approach by manipulating the magnitude of the change, allowing the researchers to identify properties of memory performance as predicted by VWM resource models—specifically in regard to “noisy” memory signals. That is, if memory representations have a degree of uncertainty, it would also make it more difficult to tell the difference between close colors than for far colors. Keshvari and colleagues (2013) varied the magnitude of difference in a change detection task, critically, including close-color discrimination trials. When large color changes were presented, a typical *K* estimate of 3.8 was observed; however, if only small color changes were presented, the results appeared to suggest that participants had no memory for any items at set size 6. It should not be concluded that participants had no memory representations under small change conditions; rather, it should be acknowledged that the memory signal-to-noise ratio was not large enough to detect a change. Thus, if not accounting for the magnitude of comparison, very different conclusions would be drawn from the task (see also Schurgin et al., 2020).

### The current study

To summarize, several studies have examined the flexible and strategic control of VWM resources by manipulating item priority in different ways, but these studies have tended to rely on continuous measures of VWM accuracy. These continuous measures, although consistent with neural models of population coding of features in VWM, may be difficult to distinguish from chance performance at low precision while also potentially failing to capture “weak” memory signals. Moreover, although low-priority representations should have an increase in neural noise (similar to that observed at high memory loads), if these items remain in memory, above-chance memory performance should still be observed in a two-alternative forced-choice (2AFC) task if the difference between the target and lure is large (i.e., if the signal-to-noise ratio is large enough).

Consequently, the goals of the current study were to (a) examine the viability of a forced-choice task to investigate the strategic allocation of VWM resources relative to a continuous report task; (b) examine whether a forced-choice task could provide evidence for low-resolution memory representations; and (c) examine whether flexible resource allocation was affected by changes in the signal-to-noise ratio between the target and lure. To examine these questions, participants were presented a goal-driven attentional prioritization VWM task, with two response types intermixed: continuous report and 2AFC. Critically, the magnitude of difference between the target and lure color was variable in the 2AFC condition to apply a psychometric approach to measuring the properties of the memory representation (Keshvari et al., 2013). Having difficult discrimination trials would require high memory strength just as in continuous report. Because the type of response was unknown until test and a high memory strength was needed in both response types, participants should not have changed any aspects of memory encoding or maintenance in accordance with the continuous or discrete nature of the response types. To analyze the effect of the color-comparison magnitude, responses in 2AFC trials were sorted into 5° bins to compare close-color and far-color color discriminations. We predicted that the priority manipulation would impact memory resolution, creating noisier representations of items with lower priority relative to those with higher priority, consistent with continuous resource models (Emrich et al., 2017). Thus, as the cue-validity decreases (100%, 80%, 50%, 25%, 10%), performance should reflect an increase in noise in the memory representations, resulting in proportionally lower accuracy in the 2AFC trials, consistent with continuous resource models of VWM. Importantly, we predicted that performance on this task would be dependent on the difference between the target and lure (Schurgin et al., 2020), and that these measures should be overall related to those obtained through continuous report tasks.

## Methods

This study included electroencephalogram (EEG) measures, which were a key aspect in the design of the experiment but are not reported here. This leads to some idiosyncrasies in the procedure with respect to the current research question. There was no preprint available for the EEG analysis of the data at the time of submission, but EEG methods are reported in the Supplementary Materials. Data files and analysis scripts can be found at https://osf.io/qj9ue/.

### Participants

Participants were recruited from the university population on the online recruitment platform SONA and through word of mouth. The target sample size for the EEG component of the study was 40 participants. Study recruitment took place between November 2021 until the end of term in April 2022, but was interrupted by COVID-19 restrictions on campus. All participants had normal vision, normal color vision, and no neurological conditions; they were not currently taking psychoactive medications and were all right handed. Thirty-two participants completed the experiment (six males, 26 females), with an average age of 21.13 years (range, 18–28). One participant was excluded for not disclosing a neurological condition at intake. Additionally, all participants were required to have completed two doses of a federally approved vaccine against COVID-19 and were required to wear a medical mask to participate in research on campus at the time of the study. Thus, anyone who was exempt from masking or was unvaccinated was also ineligible for the study. The resulting sample size of 31 exceeds that of previous studies using similar behavioral approaches by approximately 50%. All procedures were approved by the Bioscience Research Ethics Board of Brock University.

### Procedure

**Figure 1.**
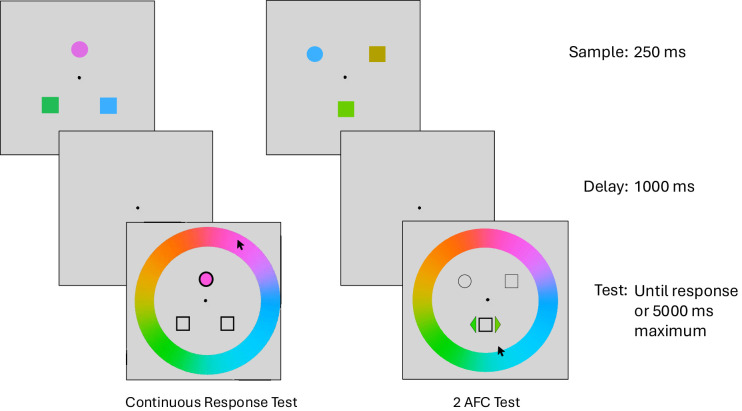
Schematic of experimental tasks. The memory sample was displayed for 250 ms, followed by a 1000-ms delay displaying only a fixation point; the test display was either continuous response (left) or 2AFC (right). The test display was terminated by response or if 5 seconds elapsed. The alternative choice in this trial was 20° away from the probe color and would be categorized into bin 2.

The experimental task consisted of two intermixed VWM response types: continuous report and 2AFC (see Figure 1). Participants were presented with three colored shapes for 250 ms (one circle and two squares) and, following a delay of 1000 ms, were asked to report one of the colors at the test phase. The unique circle distinguished which item was most likely to be probed on each trial. Participants were told the probability that the unique circle item would be tested before each block of trials. The probability that the circle would be probed was 100%, 80%, or 50%, creating three task conditions. Thus, in the 80% and 50% circle probe probability conditions, each of the two squares was tested on 10% or 25% of trials, respectively, creating five probe probability conditions. Participants were given self-paced breaks every 50 trials where they were shown performance feedback of their average absolute response error on the last block of trials. Response types were intermixed during the blocks: continuous response or 2AFC. In 100 trials per task condition, the participants’ responses were made by selecting with the mouse the color on the color wheel that best matched their memory (continuous response trials). The color would appear inside the bolded shape as participants moved the cursor around to make their selection. In the remaining trials of each condition, participants performed a 2AFC by selecting with the mouse between two colors presented on either side of the target item. One color was always the originally presented sample color, and the second color was randomly selected between ±15° and 60° away from the correct color. This distance was selected because approximately 15° is outside the range of perceptual confusion of color stimuli (Schurgin et al., 2020). Although the responses were not speeded, there was a maximum time of 5 seconds to encourage the pace of the experiment. Anecdotally, a color 60° away from the target color could be categorically unique although still confusable in poor memory conditions; for example, the far-color trials could have been a choice between blue and green. Far-color comparisons are expected to be easier than close-color comparisons, which should be demonstrated in higher accuracy and faster reaction times. The color wheel was presented in forced-choice trials (see Figure 1).

The length of the task was a maximum of 1665 trials but was intended to be variable length in order to maximize the number of trials that could be completed in the scheduled experimental session. This was done in order to maximize power for the EEG analysis not included here. Further details explaining this can be found in the Supplementary Materials. Participants were given breaks at a maximum of every 50 trials, or when all trials of a specific trial type were complete. The full experiment contained up to 1665 trials consisting of 390 trials of 100% cued probe probability, 495 trials of 80% cued probe probability, and, finally, 780 trials of 50% cued probe probability. For a breakdown of trials by response type and cue validity, see Table 1. An error in the construction of the 50% condition split meant that the actual probe probability of this condition was 48%, with the uncued items being probed 26% of the time each. On average, 1572 trials were completed by each participant; 19 participants completed all trials. The remaining 13 participants completed between 1100 and 1600 trials, with an average of 1426 trials, within the allocated time of the experiment. The very low number of 10% probe probability continuous response trials limits the accuracy of the response distribution of this condition (12–19 trials).

**Table 1.** Trials per condition by response type.

	Continuous report		2AFC		
Target probe probability	Cued	Uncued	Cued	Uncued	Total
100%	100	N/A	290	N/A	390
80%	80	19	316	80	495
50%	49	51	326	354	780

All participants completed practice trials before the experimental session which included both types of responses (continuous and 2AFC) and all three attention allocation conditions. The practice session condition order was controlled such that the 100% condition with the continuous response was first. After the first five trials, the 2AFC trials were intermixed with the continuous response. Practice trials continued with the 100% condition, followed by the 80% condition and then the 50% condition. This order was chosen to facilitate learning of the conditions and response screens. When participants began, they were not informed of the limited response window but always discovered it during the practice trials, where it was explained verbally. Participants were given the opportunity to ask questions or clarify the instructions.

### Stimuli and apparatus

Stimuli were presented using PsychoPy 2021.2.3 (Peirce et al., 2019), on a 20-inch LCD2090UXi computer monitor (1600 × 1200 pixels, 60-Hz refresh rate; NEC, Tokyo, Japan) at a distance of approximately 57 cm. The monitor was calibrated using a CS-100A Luminance and Color Meter (Konica Minolta, Tokyo, Japan). Three colored shapes (one circle, two squares) of 1° of visual angle were evenly spaced around the central fixation during the study phase. The relative spacing was consistent, but the shapes could appear among six possible locations. Thus, there were two overall configurations: clock hour-hand positions of 12:00, 4:30, and 7:30 or 1:30, 6:00, and 10:30 (see Figure 1). Colors were pseudorandomly selected from 360 unique colors from the CIE L*a*b* color space calibrated to the monitor and lighting conditions (L = 70, a = −6, b = 14; radius = 49) with a minimum distance of 30°. A color wheel with a radius 7° visual angle was presented during the color judgment phase of all conditions.

### Analysis

#### Continuous response

Error on continuous response trials was calculated as the circular distance between the target color and response color. Trials that timed out were excluded from analysis. Response precision was calculated as the circular standard deviation using the *circular* package in R (Agostinelli & Lund, 2023). Error distribution graphs are based on raincloud plots showing the shape of the density distribution along with the individual trial data below (Allen, Poggiali, Whitaker, Marshall, & Kievit, 2021).

##### Model fitting

To examine the presence of flat versus circular distributions with a central tendency, recall that errors in the 10% priority were examined for each subject using MemToolbox (Suchow, Brady, Fougnie, & Alvarez, 2013) in MATLAB R2021a (MathWorks, Natick, MA). Errors were fit to two models, the “all guessing” model (i.e., a uniform distribution) and the “standard mixture” model, with the guess parameter fixed at 0 (i.e., a circular normal distribution). They were compared using the Bayesian information criterion (BIC). The 2AFC data were then compared against chance (0.5, one-tailed) using JASP (JASP Team, 2024).

#### Two-alternative forced-choice

##### Accuracy

To model the parametric effect of probe probability on response accuracy, the 2AFC data were split into nine bins depending on the magnitude of the difference between the lure and the probe color (discrimination bins). Each bin represented 5° of circular color deviations, starting with the closest color comparison of 15° to 19°, and so on, except for the ninth bin, which covered 55° to 60°. The proportion correct data in 2AFC tasks can be adjusted for guessing (chapter 7, Hautus, Macmillan, & Creelman, 2022) using the formula:

q=(2×proportioncorrect)-1

The adjusted proportion correct for each bin within each probe probability was used as the measure of accuracy for analysis. Trials that timed out were excluded from analysis. Because the accuracy data were adjusted for guessing, chance performance would be a score of zero, thus one-sample *t*-tests were used to test performance differing from chance.

To test the effect of probe probability and discriminability of lure distance on 2AFC performance, accuracy (adjusted proportion correct) was modeled with a linear mixed-effect model with a random intercept for each participant and fixed effects of the predictor variables of discrimination bins and probe probability conditions, as well as the potential interaction. Both probe probability and discrimination bins were treated as continuous variables in the mixed-effect model. Models were performed using the *lme4* package in R (Bates, Maechler, Bolker, & Walker, 2015), and ANOVA (type 2) from the *car* package in R (Fox & Weisberg, 2019).

### Comparing performance by response type

Additionally, an exploratory analysis was performed to compare the performance between delayed-estimation and 2AFC trials by analyzing the 2AFC responses as if they were continuous responses. This analysis demonstrates the relationship between the response types. Response precision (circular standard deviation) was compared for each participant. For 2AFC responses, response error could be calculated in the same manner as the continuous responses due to the variable magnitude of the target–lure distance. That is, “hits” always had an error of 0°, whereas “misses” could be between 15° and 60°. The standard deviation of the error was calculated on an assumed circular normal distribution, although the actual distribution of responses was not normal due to the density at 0° error and the gap between 1° and 14° of error (see Supplementary Figure S1 for the error distribution of 2AFC data by probe probability condition). Thus, the exact standard deviation values of 2AFC responses should not be interpreted as precise values, as they are systematically skewed; however, for comparison to continuous response trials, the pattern of change and relationship to the conditions can be interpreted. As well, the very low number of 10% probe probability continuous response trials limits the accuracy of interpreting the standard deviation of this condition and response type (12–19 trials). However, comparing the overall pattern of performance between the response types is more important for this experimental analysis. The data were fit to a Bayesian linear mixed-effect model using the *blme* package in R (Chung, Rabe-Hesketh, Dorie, Gelman, & Liu, 2013).

## Results

### Precision and accuracy

This study was designed to (a) examine the viability of a forced-choice task to investigate the strategic allocation of VWM resources relative to a continuous report task, (b) examine whether a forced-choice task could provide evidence for low-resolution memory representations, and (c) examine whether flexible resource allocation was affected by changes in the signal-to-noise ratio between the target and lure. The study combined continuous responses and 2AFC response types, and a shape cue indicated probe probability. Manipulating probe probability has been demonstrated previously to be comparable to manipulations of set size (Emrich et al., 2017).

**Figure 2.**
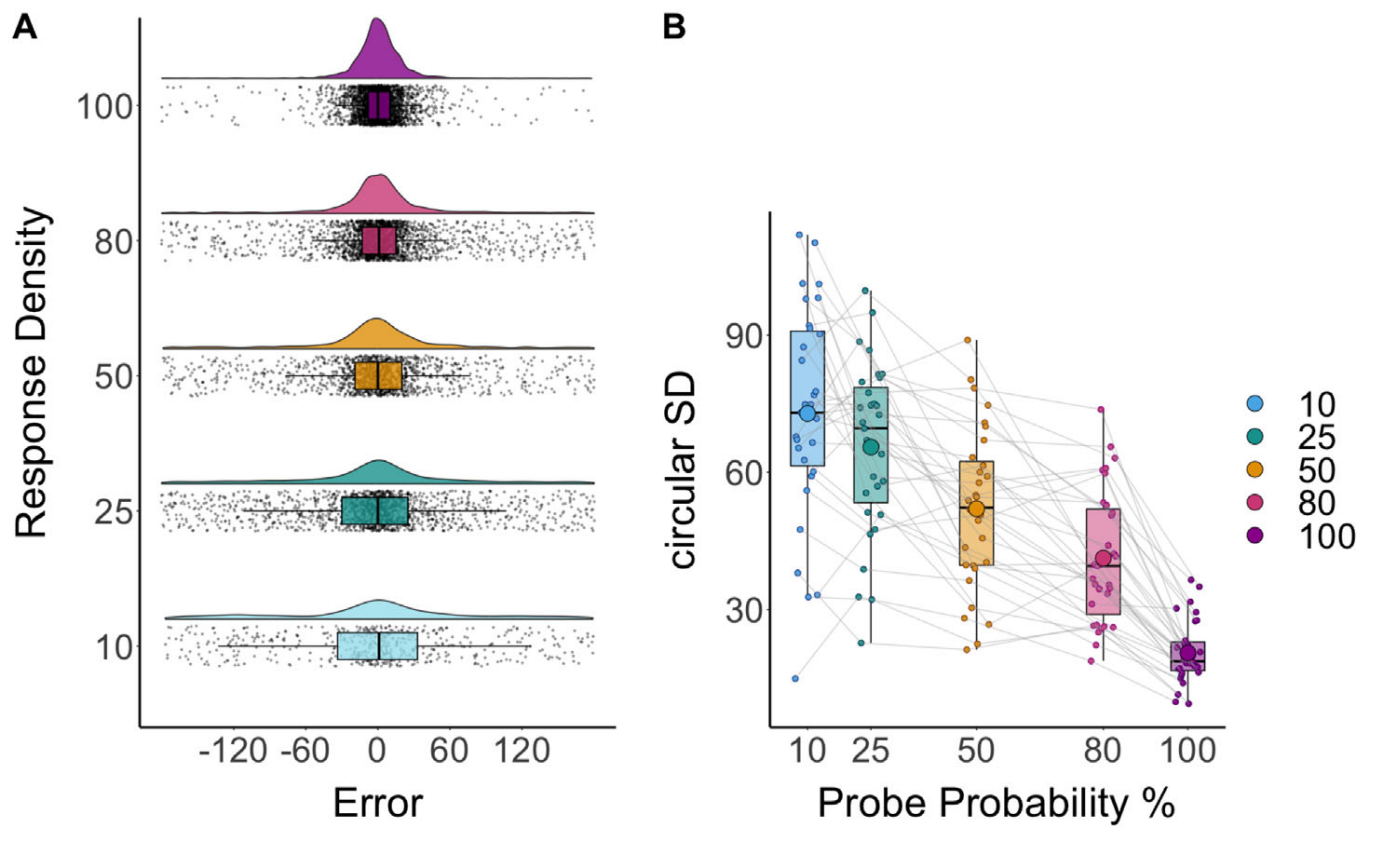
Continuous report performance by probe probability conditions. (**A**) Response density distributions of error from continuous report data for each probe probability condition. Box plots are overlaid on all participants’ datapoints. (**B**) Circular standard deviation (1/precision) by probe probability conditions for continuous report data.

To establish that the priority manipulation worked similarly in this study as in past studies, the response error of the continuous report trials was analyzed. As in previous studies, error in continuous report trials was lower at higher probe probabilities, suggesting greater resource allocation to these items in a flexibly allocated manner (Dube et al., 2017; Emrich et al., 2017) (see Figure 2). Moreover, the response error (as measured by circular standard deviation) increased continuously as the proportion of resources allocated to a probed item decreased, consistent with a flexible and continuous resource (e.g., Emrich et al., 2017).

**Figure 3.**
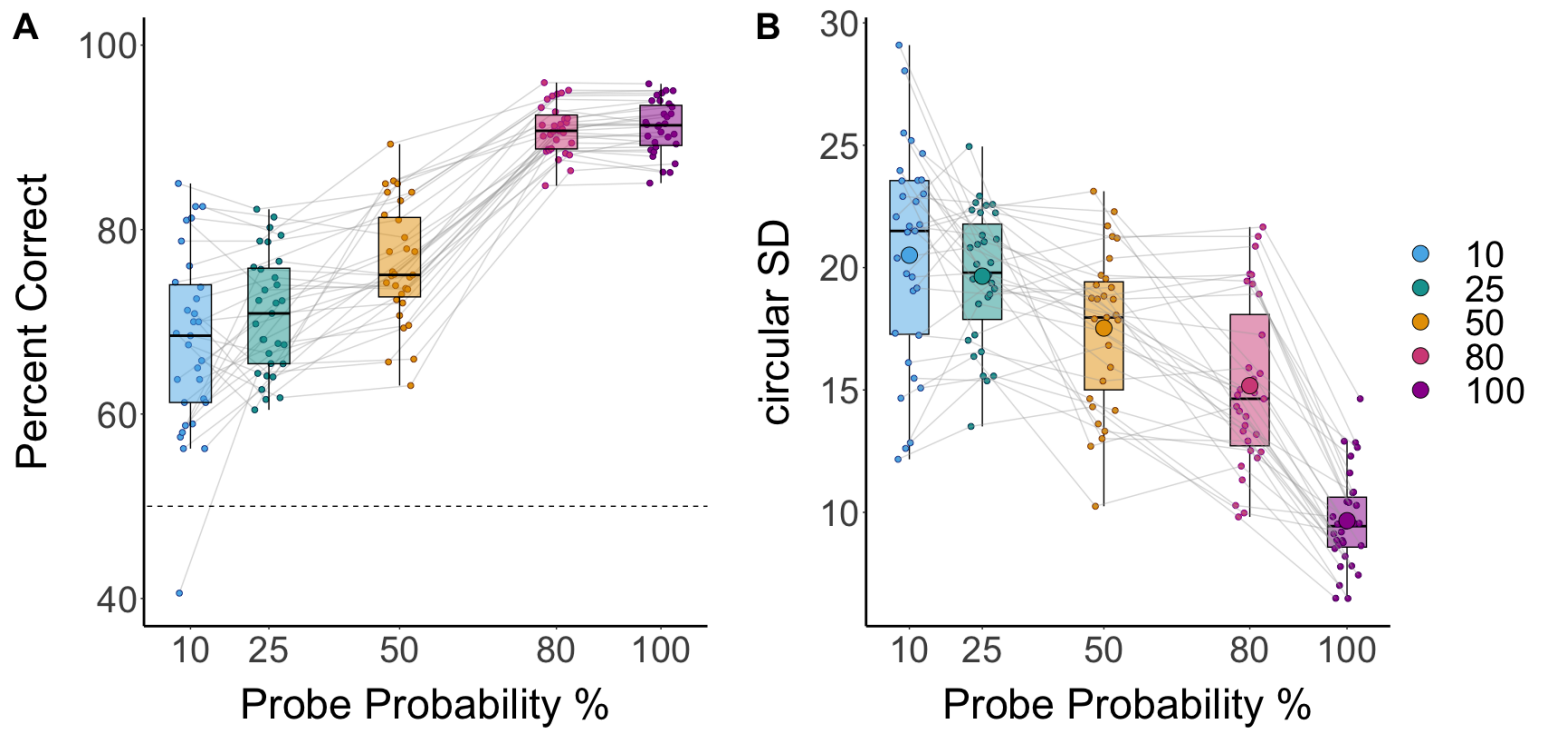
2AFC performance by probe probability conditions. (**A**) Percent correct by probe probability of 2AFC trials; participant data points and a chance performance line are shown. (**B**) Circular standard deviation (precision) by probe probability for the 2AFC responses with a box plot overlaid; individual participants’ data are shown; mean performance is indicated by large circles.

Similarly, consistent with studies that manipulate set size, response accuracy in 2AFC trials (percent correct) decreased when fewer resources were allocated to the item, *F*(4, 120) = 187.98, *p* < 0.001 (see Figure 3). Notably, even in the lowest probe probability condition, participants were an average of 68% accurate, and all but one performed above chance.

**Figure 4.**
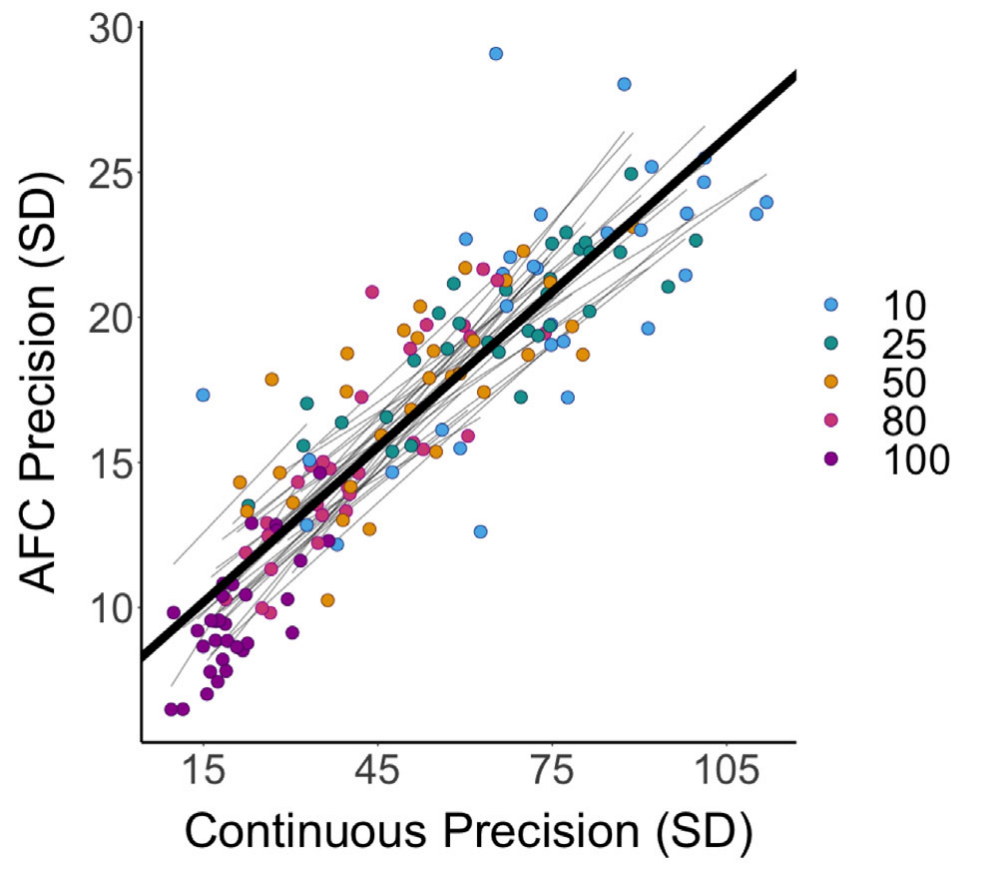
Comparison of circular standard deviation performance between response type. Individual participant linear fits are shown in gray lines. The black line shows the linear fit from the Bayesian linear mixed-effect model with the average intercept for graphical purposes.

To demonstrate that the continuous data and 2AFC data do not reflect completely different aspects of memory performance, the responses for each condition by each response type were compared within each participant. Figure 4 shows the significant linear correlation between performance (circular standard deviation of response error) on continuous report trials and 2AFC trials, *F*(1, 153) = 548.27, *p* < 0.001. This relationship confirmed that participant performance across the two response types was conserved, such that high performance on the 2AFC trials was associated with high performance on the continuous report trials.

### Psychometrics of 2AFC performance

**Figure 5.**
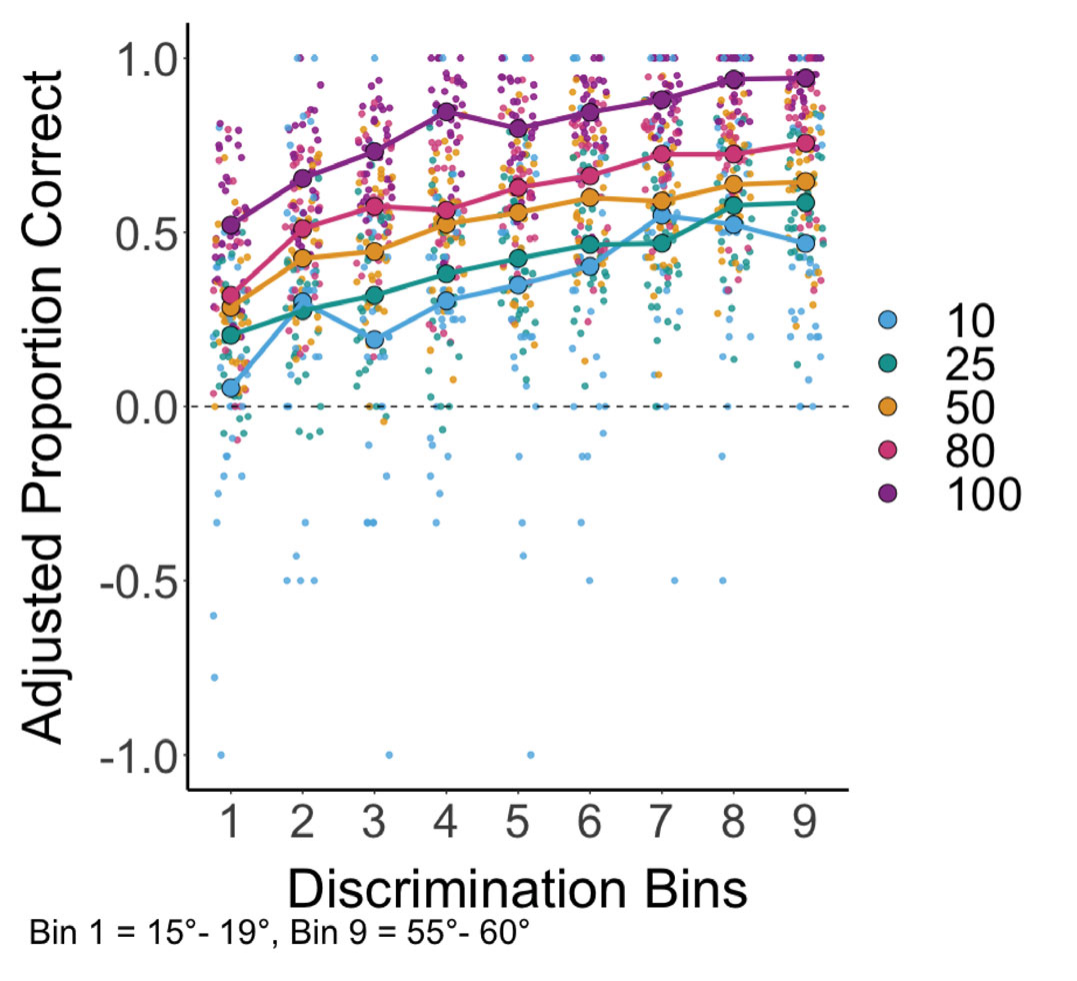
2AFC performance measures by decision difficulty. The adjusted proportions correct by discrimination bins (close-color bin 1, 15°–19° difference; far-color bin 9, 55°–60° difference) are shown for the probe probability conditions; individual participants’ data are indicated in the small circles and means in the large circles. The chance performance line is shown.

Drawing on the framework of signal detection theory, predictions about response error can be investigated in more detail by analyzing the impact of task difficulty on performance in conjunction with the probe probability manipulation (see Supplemental Materials for a similar analysis on reaction time). The 2AFC trials were grouped into nine discrimination bins based on the magnitude of the difference between the alternative choice color and the probed target color. Bin 1 included the most difficult close-color comparisons between 15° and 19°, and bin 9 included the easiest comparisons of far colors between 55° and 60°. The accuracy data were adjusted for guessing (Hautus et al., 2022). Linear mixed-effects modeling analyzes the effects of the attention manipulation (probe probability) and decision difficulty (discrimination bin) on accuracy (adjusted proportion correct). The model was built in four steps beginning with the random-intercept-only model. By adding predictors in this way, the unique effect of each predictor could be demonstrated. The best-fitting model was one that included both bin and probe probability as fixed-effect predictors, but not their interaction (see Table 2). In the best-fitting model, accuracy was predicted by fixed effects of probe probability, where Accuracy_adjusted proportion_ ≈ discriminability_bin_ + probe probability + (1|participantID) (β = 0.0046, *SE* = 0.0002, *t* = 26.83, *p* < 0.0001), and the discrimination bin (β = 0.0467, *SE* = 0.0022, *t* = 21.05, *p <* 0.001), such that the adjusted proportion correct increased by 0.37 from the close-color comparisons (bin 1) to the far-color comparisons (bin 9). The effect of discrimination bin on response accuracy was not congruent with any model of VWM resources that does not allow for variability in memory quality (e.g., item-based discrete models), but was predicted by all continuous resource models. Furthermore, accuracy increased with greater probe probability, such that the 100% condition had an adjusted proportion correct 0.23 higher than in the 50% probe probability condition. Figure 5 shows that each predictor affected accuracy without any evident interaction; accuracy increased as the task became easier between discrimination bins 1 and 9 in all probe probability conditions, and accuracy increased from the lowest probe probability (10%) up to the highest (100%) probe probability conditions.

**Table 2.** Summary of mixed-effect models of 2AFC accuracy.

Fixed effects	Random effect	χ^2^(*df*), *p*
Intercept only	Intercept per participant	—
Accuracy ≈ Discriminability_bin_	Intercept per participant	263.2 (1), **<0.001**
Accuracy ≈ Discriminability_bin_ + probe probability	Intercept per participant	578.9 (1), **<0.001**
Accuracy ≈ Discriminability_bin_ × probe probability	Intercept per participant	0.303 (1), 0.582

Significant *p* values bolded.

One characteristic difference between continuous resource models and discrete resource models is whether there are true guess responses caused by not having the test item in memory or whether there can be very low-resolution memory representations that produce very low-precision responses. To assess whether performance in any condition reflected random guessing, the accuracy data were compared to chance performance. Because the accuracy data were adjusted for guessing, performance scores were compared to 0 instead of 50% (see Figure 5). Accuracy in the lowest probe probability condition (10%) at the closest color discrimination bin (15°–19°) was not different from chance; however, performance was better than chance for the next discrimination bin (20°–24°) and every discrimination bin thereafter. For all other probe probability conditions at every level of discrimination, accuracy was above chance.

#### Do 2AFC responses capture low-resolution representations?

As outlined in the Introduction, one of the limitations of continuous response tasks is that it is often difficult to distinguish responses that predominantly reflect items that are outside of memory (i.e., guesses) versus representations that are in memory but which have very low-resolution responses. To examine whether the 2AFC task could better capture low-resolution memories, we fit the continuous response data in the 10% probability condition to two models, such that the flat, uniform distribution (i.e., one that reflects pure guesses) model fit was compared to that of a circular normal distribution. Although the group-averaged data in this condition appear to demonstrate a central tendency (Figure 2A, bottom), the data of 14 of the 31 participants (45%) were better fit to a uniform distribution (∆BIC = −3.0), with one additional participant demonstrating a nearly equivalent fit (∆BIC = 0.03). However, despite the lack of evidence of representations in these distributions, the 2AFC performance in these participants was significantly above chance, µ = 0.62, *t*(13) = 8.514, *p* < 0.001, Cohen's *d* = 2.275. Thus, although continuous response distributions are difficult to distinguish between pure guessing and low-resolution memories, performance on 2AFC tasks provides strong evidence in favor of low-resolution memories, even at very minimal resource allocation.^1^

## Discussion

The investigation of low-precision memory representations is a critical point of distinction between discrete and continuous resource models in VWM. Because VWM is capacity limited, the allocation of a small proportion of total resources (through either increasing memory load or attentional prioritization) should result in low-resolution memory representations. When measured through error distributions from delayed-estimation paradigms, it can be difficult to distinguish the expected behavior from reporting a low-resolution representation (a resulting wide and flat error distribution) from the expected behavior of an out-of-memory guess response (a resulting uniform error distribution). Moreover, increases in memory load will both decrease the signal (resulting in these low-resolution memories) and increase the noise, thus making it more difficult to assess these low-resolution representations.

Consequently, the current study assessed memory for low-resolution items as determined by attentional prioritization using both continuous and discrete responses in an intermixed design. We predicted that if low-resolution items are in memory, consistent with continuous resource models, memory performance for these items should be above chance in the forced-choice trials, even with very flat distributions in the delayed-estimation trials. Consistent with previous studies (Dube et al, 2017; Emrich et al., 2017), the precision of memory report in the delayed-estimation task was directly related to the likelihood that a given item would be probed, consistent with the flexible allocation of continuous resources.

Critically, during the recognition memory task, participants were better than chance at selecting the target color, even in the most difficult color discrimination condition at low task relevance (25% probe probability and 15°–20° of color separation), and they were better than chance at all but the most difficult discriminations at very low task-relevant items (10% probe probability and 20°–25° of color separation). Thus, even at very low resource allocation (equivalent to an item load of 10 in a traditional memory task without cues) (Emrich et al., 2017), evidence of memory for the 10% items in all but the smallest discrimination differences provides compelling evidence that these items are represented in memory. Moreover, even in those participants whose data were best described by a flat (uniform) distribution in the continuous response task (typically interpreted as indicative of pure guessing), the participants still exhibited above-chance performance in the equivalent 2AFC trials. Evidence from this recognition memory task supports a model of VWM with low-resolution memory representations that can be lost in a noisy signal and look like guessing under less sensitive paradigms. This evidence is in line with signal detection frameworks of VWM which are in turn supported by plausible neurophysiological mechanisms of neural population coding with noise (Bays, 2014; Bays, 2015).

Importantly, performance (precision) on the continuous response and 2AFC trials was strongly correlated, such that high performance in one response type predicted high performance in the other. This finding supports a conclusion that the underlying memory representations are the same and do not depend on the type of response used to assess those representations. These data provide further support for continuous resource models by demonstrating again that response precision is predicted by probe probability but in a paradigm that has not been used to test this theory before. It is noted, however, that the circular standard deviation of the 2AFC data carries the significant caveat that it is not normally distributed.

Further support for the predictions of a continuous resource model comes from the analysis of accuracy across differences in the difficulty of discrimination for each level of probe probability. As expected by continuous models of memory resources, there is a main effect of task difficulty such that the close-color choices had lower accuracy than the far-color choices. As highlighted by van den Berg and colleagues (2012) with respect to change localization, the classic item-limit discrete resource model does not have any flexibility to accommodate an effect of variable difficulty on the accuracy of discrimination because of an assumed noiseless memory representation. Instead, this effect supports a resource model with noisy memory representations.

Crucially, probe probability led to graded differences in response accuracy consistent with predicted changes to the signal-to-noise ratio of the memory representations. That is, although the closer color comparisons were more difficult, higher allocation of VWM resources allowed participants to correctly distinguish the target color from memory. This suggests that the priority of the item (i.e., task relevance) had a direct effect on memory quality, presumed to be the effect of goal-directed attention influencing resource allocation—in other words, the flexible allocation of memory resources.

This psychometric approach to the discrete response data further bolsters the predictions of continuous resource models by not requiring the comparison of error distribution parameters. Instead, the accuracy data can be analyzed over controlled changes in difficulty. This is an important extension of the application of continuous resource models because it demonstrates support for the predictions of a theoretic continuous resource in a paradigm other than delayed-estimation. In addition to the evidence from change detection (Keshvari et al., 2013; Pearson et al., 2014), and change localization (van den Berg et al., 2012), there is clear support for continuous resource models regardless of whether or not memory precision can be directly measured through error distributions.

The intermixed design of the experiment discouraged participants from differently encoding the color stimuli on a trial-by-trial basis based on the response type; however, due to the higher proportion of 2AFC trials, it is possible that participants could have used a different strategy than if all of the trials had been delayed-estimation continuous reports. Because the 2AFC trials did not use maximally different lures and did include difficult close-color comparisons, 2AFC trials should still have required a high memory strength.

Although the results of this study align best with the predictions of continuous resource models, the predictions of any specific continuous resource model were not tested. As well, some results could be explained by flexible variations of discrete resource models which was not ruled out by direct testing. Overall, the data are best conceptualized as coming from a noisy memory representation that can be improved with greater priority given to the item in a flexible manner. Importantly, these results did not depend on any model estimated parameters, instead comparing conditions on the model-free summary statistics instead (Ma, 2018).

## Conclusions

These data demonstrate that flexible allocation of a continuous memory resource is evident beyond continuous response tasks and support the underlying signal detection framework of continuous resource models. The 2AFC data provided evidence that flexible allocation of fewer memory resources results in a noisier memory representation, leading to lower accuracy as predicted by a signal detection framework. Critically, above-chance performance of the lowest probe probability condition across difficulty conditions suggests that responses to these items reflect low-resolution representations rather than guesses, which could be inferred from the wide error distributions from continuous responses. Thus, these data support frameworks in which even low-resolution items, when assessed properly, are stored in memory. The combination of a 2AFC task and attentional prioritization used here provides a mechanism to assess low-memory signal strength while minimizing the amount of noise, thus revealing new evidence for the existence of these low-precision memory representations.

## Supplementary Material

Supplement 1

## Footnotes

1 It is worth noting that it is possible that the relatively small number of trials in the continuous response task resulted in a uniform distribution in these individuals and that an equal distribution of trials across conditions may produce a different result. Nevertheless, these results provide proof of concept that the 2AFC task can produce above-chance performance even when the continuous response distributions are difficult to differentiate from a pure-guessing model.

## References

[bib1] Adam, K. C. S., Vogel, E. K., & Awh, E. (2017). Clear evidence for item limits in visual working memory. *Cognitive Psychology,* 97, 79–97, 10.1016/j.cogpsych.2017.07.001. 28734172 PMC5565211

[bib2] Alvarez, G. A., & Cavanagh, P. (2004). The capacity of visual short-term memory is set both by visual information load and by number of objects. *Psychological Science,* 15(2), 106–111, 10.1111/j.0963-7214.2004.01502006.x. 14738517

[bib3] Agostinelli, C., & Lund, U. (2023). circular: Circular Statistics. Retrieved from cran.r-project.org/package=circular.

[bib4] Allen, M., Poggiali, D., Whitaker, K., Marshall, T. R., & Kievit, R. A. (2021). Raincloud plots: A multi-platform tool for robust data visualization. *Wellcome Open Research,* 4, 63, 10.12688/wellcomeopenres.15191.1. 31069261 PMC6480976

[bib5] Bates, D., Maechler, M., Bolker, B., & Walker, S. (2015). Fitting linear mixed-effects models using lme4. *Journal of Statistical Software,* 67(1), 1–48, 10.18637/jss.v067.i01.

[bib6] Bays, P. M. (2014). Noise in neural populations accounts for errors in working memory. *The Journal of Neuroscience,* 34(10), 3632–3645, 10.1523/jneurosci.3204-13.2014. 24599462 PMC3942580

[bib7] Bays, P. M. (2015). Spikes not slots: Noise in neural populations limits working memory. *Trends in Cognitive Sciences,* 19(8), 431–438, 10.1016/j.tics.2015.06.004. 26160026

[bib8] Bays, P. M., Catalao, R. F. G., & Husain, M. (2009). The precision of visual working memory is set by allocation of a shared resource. *Journal of Vision,* 9(10):7, 1–11, 10.1167/9.10.7. PMC3118422 19810788

[bib9] Bays, P. M., & Husain, M. (2008). Dynamic shifts of limited working memory resources in human vision. *Science,* 321, 851–854, 10.1126/science.1158023. 18687968 PMC2532743

[bib10] Brady, T. F., Robinson, M. M., Williams, J. R., & Wixted, J. T. (2023). Measuring memory is harder than you think: How to avoid problematic measurement practices in memory research. *Psychonomic Bulletin & Review,* 30(2), 421–449, 10.3758/s13423-022-02179-w. 36260270 PMC10257388

[bib11] Chung, Y., Rabe-Hesketh, S., Dorie, V., Gelman, A., & Liu, J. (2013). A nondegenerate penalized likelihood estimator for variance parameters in multilevel models. *Psychometrika,* 78(4), 685–709, 10.1007/s11336-013-9328-2. 24092484

[bib12] Dube, B., Emrich, S. M., & Al-Aidroos, N. (2017). More than a filter: Feature-based attention regulates the distribution of visual working memory resources. *Journal of Experimental Psychology: Human Perception and Performance,* 43(10), 1843–1854, 10.1037/xhp0000428. 28967787

[bib13] Emrich, S. M., Lockhart, H. A., & Al-Aidroos, N. (2017). Attention mediates the flexible allocation of visual working memory resources. *Journal of Experimental Psychology: Human Perception and Performance,* 43(7), 1454–1465, 10.1037/xhp0000398. 28368161

[bib14] Fox, J., & Weisberg, S. (2019). *An R companion to applied regression* (3rd ed.). Thousand Oaks, CA: Sage College Publishing.

[bib16] Hautus, M. J., Macmillan, N. A., & Creelman, C. D. (2022). *Detection theory: A user's guide* (3rd ed.). New York: Routledge, 10.4324/9781003203636.

[bib17] Huynh Cong, S., & Kerzel, D. (2022). The allocation of working memory resources determines the efficiency of attentional templates in single- and dual-target search. *Journal of Experimental Psychology: General,* 151(12), 2977–2989, 10.1037/xge0001239. 35696178

[bib18] JASP Team. (2024). JASP 0.19.2. Retrieved from https://jasp-stats.org/previous-versions/.

[bib19] Keshvari, S., van den Berg, R., & Ma, W. J. (2013). No evidence for an item limit in change detection. *PLoS Computational Biology,* 9(2), e1002927, 10.1371/journal.pcbi.1002927. 23468613 PMC3585403

[bib20] Klyszejko, Z., Rahmati, M., & Curtis, C. E. (2014). Attentional priority determines working memory precision. *Vision Research,* 105, 70–76, 10.1016/j.visres.2014.09.002. 25240420 PMC4250278

[bib21] Luck, S. J., & Vogel, E. K. (1997). The capacity of visual working memory for features and conjunctions. *Nature,* 390(6657), 279–281, 10.1038/36846. 9384378

[bib21a] Luck, S. J., & Vogel, E. K. (2013). Visual working memory capacity: From psychophysics and neurobiology to individual differences. *Trends in Cognitive Sciences,* 17(8), 391–400, 10.1016/j.tics.2013.06.006. 23850263 PMC3729738

[bib22] Ma, W. J. (2018). Problematic usage of the Zhang and Luck mixture model. bioRxiv, 10.1101/268961.

[bib23] Ma, W. J., Husain, M., & Bays, P. M. (2014). Changing concepts of working memory. *Nature Neuroscience,* 17(3), 347–356, 10.1038/nn.3655. 24569831 PMC4159388

[bib25] Oberauer, K., Lewandowsky, S., Awh, E., Brown, G. D. A., Conway, A., Cowan, N., … Ward, G. (2018). Benchmarks for models of short-term and working memory. *Psychological Bulletin,* 144(9), 885–958, 10.1037/bul0000153. 30148379

[bib26] Oberauer, K., & Lin, H.-Y. (2017). An interference model of visual working memory. *Psychological Review,* 124(1), 21–59, 10.1037/rev0000044. 27869455

[bib27] Pashler, H. (1988). Familiarity and visual change detection. *Attention, Perception, & Psychophysics,* 44(4), 369–378, 10.3758/BF03210419. 3226885

[bib28] Pearson, B., Raskevicius, J., Bays, P. M., Pertzov, Y., & Husain, M. (2014). Working memory retrieval as a decision process. *Journal of Vision,* 14(2):2, 1–15, 10.1167/14.2.2. PMC3912875 24492597

[bib29] Peirce, J., Gray, J. R., Simpson, S., MacAskill, M., Höchenberger, R., Sogo, H., … Lindeløv, J. K. (2019). PsychoPy2: Experiments in behavior made easy. *Behavior Research Methods,* 51(1), 195–203, 10.3758/s13428-018-01193-y. 30734206 PMC6420413

[bib30] Salahub, C., Lockhart, H. A., Dube, B., Al-Aidroos, N., & Emrich, S. M. (2019). Electrophysiological correlates of the flexible allocation of visual working memory resources. *Scientific Reports,* 9, 19428, 10.1038/s41598-019-55948-4. 31857657 PMC6923388

[bib31] Schurgin, M. W. (2018). Visual memory, the long and the short of it: A review of visual working memory and long-term memory. *Attention, Perception, & Psychophysics,* 80(5), 1035–1056, 10.3758/s13414-018-1522-y. 29687357

[bib32] Schurgin, M.W., Wixted, J.T. & Brady, T.F. (2020). Psychophysical scaling reveals a unified theory of visual memory strength. *Nature Human Behavior,* 4(11), 1156–1172, 10.1038/s41562-020-00938-0. 32895546

[bib34] Suchow, J. W., Brady, T. F., Fougnie, D., & Alvarez, G. A. (2013). Modeling visual working memory with the MemToolbox. *Journal of Vision,* 13(10):9, 1–8, 10.1167/13.10.9. PMC4521709 23962734

[bib35] van den Berg, R., & Ma, W. J. (2018). A resource-rational theory of set size effects in human visual working memory. *eLife,* 7, e34963, 10.7554/eLife.34963. 30084356 PMC6110611

[bib36] van den Berg, R., Shin, H., Chou, W.-C., George, R., & Ma, W. J. (2012). Variability in encoding precision accounts for visual short-term memory limitations. *Proceedings of the National Academy of Sciences, USA,* 109(22), 8780–8785, 10.1073/pnas.1117465109. PMC3365149 22582168

[bib37] Wilken, P., & Ma, W. J. (2004). A detection theory account of change detection. *Journal of Vision,* 4(12):11, 1120–1135, 10.1167/4.12.11. 15669916

[bib38] Yoo, A. H., Klyszejko, Z., Curtis, C. E., & Ma, W. J. (2018). Strategic allocation of working memory resource. *Scientific Reports,* 8, 16162, 10.1038/s41598-018-34282-1. 30385803 PMC6212458

[bib39] Zhang, W., & Luck, S. J. (2008). Discrete fixed-resolution representations in visual working memory. *Nature,* 453, 233–235, 10.1038/nature06860. 18385672 PMC2588137

