# Supplementary material for: Low-priority items are held in visual working memory: Evidence from flexible allocation in a two-alternative forced-choice (2AFC) paradigm
Source: J Vis. 2025 Mar 7;25(3):5. doi: 10.1167/jov.25.3.5 (PMC11905579; doi:10.1167/jov.25.3.5)
Supplement: Supplement 1 [file jovi-25-3-5_s001.pdf]

Supplemental Methods

Participants

Participants had to meet inclusion criteria such as being right-handed, no history of head injury, no neurological conditions, no cognitively active medications. Participants completed an intake form on Qualtrics to ensure that they met the inclusion criteria before scheduling.

Apparatus

EEG Recordings were taken using the Biosemi Active-2 system. Participants were fit with 64 channel EEG caps using extended 10-20 sites plus 6 exogenous electrodes placed on each mastoid bone, laterally beside the eyes, and below each eye.

Procedure

The length of task was designed to maximize the number of trials within the scheduled three-hour experimental session (while accounting for EEG set-up, practice, clean-up, etc. with the participant) thus it was anticipated that many participants would not complete all of the trials. As such, the three task conditions were presented in blocks of up to 50 trials at a time, with a random order so that the conditions were roughly maintained regardless of the stopping time. The trials within the blocks were pseudo-randomly assigned so that the cued and uncued trials were consistent with the instruction of the condition (i.e., a block of 80% probe probability trials would not have 50% uncued trials). The full experiment had 1665 trials, if each trial lasted 5 seconds the task would take 2 hours and 19 minutes leaving approximately 40 minutes for consent forms, EEG set-up, instruction and practice, cap removal and clean-up, and self-paced breaks between the experimental blocks. A critical aspect of the study design was that conditions were constructed to have 60-65 trials with the target in each of the 6 possible target locations for an EEG analysis not included here. Due to this construction the final block of trials in each

condition was not a full 50 trials. The full experiment contained up to 1665 trials consisting of 390 trials of 100% cued probe probability, 495 trials of 80% cued probe probability, and lastly 780 trials 50% cued probe probability. For breakdown of trials by response type and cue validity see Table 1. Notably there were 99-100 continuous report trials total per condition with the remaining trials were 2AFC. A deciding factor to not have equivalent trials of continuous report and 2AFC is that 2AFC trials are typically faster than continuous report and would allow for more trials to be completed per experimental session, as well as to allow enough trials to conduct an analysis on different target-lure distance bins. Thus, only the minimum number of continuous report trials were completed per condition in order to construct reliable response distributions comparable to past studies (e.g. Emrich et al., 2017).

Supplemental Results

In addition to the effects of accuracy described in the main texts, greater noise in the memory representations should also influence response time (RT). Response time (RT) provides information about the decision processes utilized during VWM paradigms. A typical model of decision making proposes the accumulation of information up to a decision threshold. In sequential sampling signal detection frameworks, decision time (and thus RT) is longer when the information that is sampled is noisier because it takes longer for evidence to reach the threshold (Noorani & Carpenter, 2016; Smith & Ratcliff, 2009). This can be exemplified by Pearson and colleagues (2014), in a robust study of 4,680 change detection trials in three participants it was found that memory quality impacted the rate of evidence accrual and not the decision threshold. Manipulations of memory quality (for instance, using task-relevance cues) had predictable corresponding changes in RT (Pearson et al., 2014). It was predicted that as the cue-validity decreases (100%, 80%, 50%, 25%, 10%) performance should reflect having more noise in the memory representations, which in addition to the effects on accuracy would also result in longer RTs due to slower rate of evidence accrual.

Response Time Analysis

Response time data were trimmed using the *trimr* package (Grange, 2022), using an upper criterion of 2.5 SD computed for each participant at each level of probe probability and discrimination bin (i.e., 10% probe probability and 15-19°, etc.); hit and miss responses were separately trimmed. The effect of probe probability and discrimination bin on response time was tested using the same approach as described above for accuracy, with the one difference that all trials were modelled rather than a summary statistic. This difference allows for the variable number of trials per predictor category (probe probability and discrimination bins) to be reflected

in the uncertainty for each participant's model.

Response Time Results

Predictions for the RT data are derived from the sequential sampling predictions of signal detection theory: that is lower probe probability will lead to a noisier representation in memory, and then it takes longer to make a decision in noisier conditions. The RT data for hits and misses were separately trimmed with a cut-off of 2.5 SD per participant per experimental conditions (probe probability and discrimination bin), as well as a minimum time of 100ms. These data were fit to a linear mixed-effect model predicting RT from the fixed-effects of discrimination bin and probe probability, as well as their interaction with a random intercept for each participant. The full model including the interaction for hit responses was significant: $\texttt{RT}_{\texttt{hits)}} \sim \texttt{discriminability}_{\texttt{(bin)}}\ \texttt{probe probability + (1|participantID)}$, ($X^2(1) = 6.44$, $p = .0112$), see supplementary Figure S2. In general, it takes approximately 74.47 ms longer to make a correct response for close-color comparisons (bin 1) than far-color comparisons (bin 9) ($\beta = -9.309$ ms, $se = 0.795$, $t = 11.710$, $p < .0001$; estimated in 50% probe probability condition); and it takes approximately 120 ms longer to make a correct response in the 50% probe probability condition compared with the 100% probe probability condition ($\beta = -2.400$ ms, $se = 0.123$, $t = 19.498$, $p < .0001$; estimated at bin 1 representing a close-color comparison). Additionally, these effects interact ($\beta = -0.062$ ms, $se = 0.002$, $t = 2.537$, $p = 0.0112$), in the manner that the difference in RT between probe probability conditions is greater at close-color comparisons than at far color comparisons.

Supplemental Discussion

The response time analysis indicates that greater resource allocation in higher probe probability conditions led to faster reaction times. Further, this interacted with the

discriminability difficulty, such that the difference in RT between probe probability conditions was greater at the difficult close-color trials than at the easier far-color discrimination trials. This is consistent with the predictions of continuous resource models as applied by the signal detection framework. Signal detection proposes that response time indicates the time needed for evidence to accumulate to come to a decision between the two alternate choices. Easier decisions should be made faster than more difficult ones when the colors are closer together which was demonstrated by the main effect of discrimination difficulty. Secondly, decisions are predicted to be easier (and thus faster) if the memory representation is less noisy which is demonstrated by the main effect of priority condition (probe probability). The model of flexible allocation of continuous resources predicts that greater memory resource allocation results in a higher resolution memory representation, which in turn leads to faster response times. This effect interacted with discrimination difficulty such that the beneficial effect of memory quality had a greater impact on RT for difficult discrimination trials than on easier trials. Similarly, Pearson and colleagues (2014) found that both the memory quality and the discrimination difficulty interacted to affect the slope of evidence accumulation and not the decision threshold or the trial-to-trial variance in the rate of accumulation.

Further supporting the predictions of a signal detection framework of VWM resources, the 2AFC data demonstrated slower response times as probe probability decreased in line with the hypothesis that flexible allocation of fewer memory resources results in a noisier memory representations.

References

Grange, J. (2022). trimr: An implementation of common response time trimming methods. Retrieved from cran.r-project.org/package=trimr.

Noorani, I., & Carpenter, R. H. S. (2016). The LATER model of reaction time and decision. *Neuroscience & Biobehavioral Reviews*, 64, 229–251, https://doi.org/10.1016/j.neubiorev.2016.02.018.

Smith, P. L., & Ratcliff, R. (2009). An integrated theory of attention and decision making in visual signal detection. *Psychological Review,* 116(2), 283–317, https://doi.org/10.1037/a0015156.

Supplemental Figure Captions

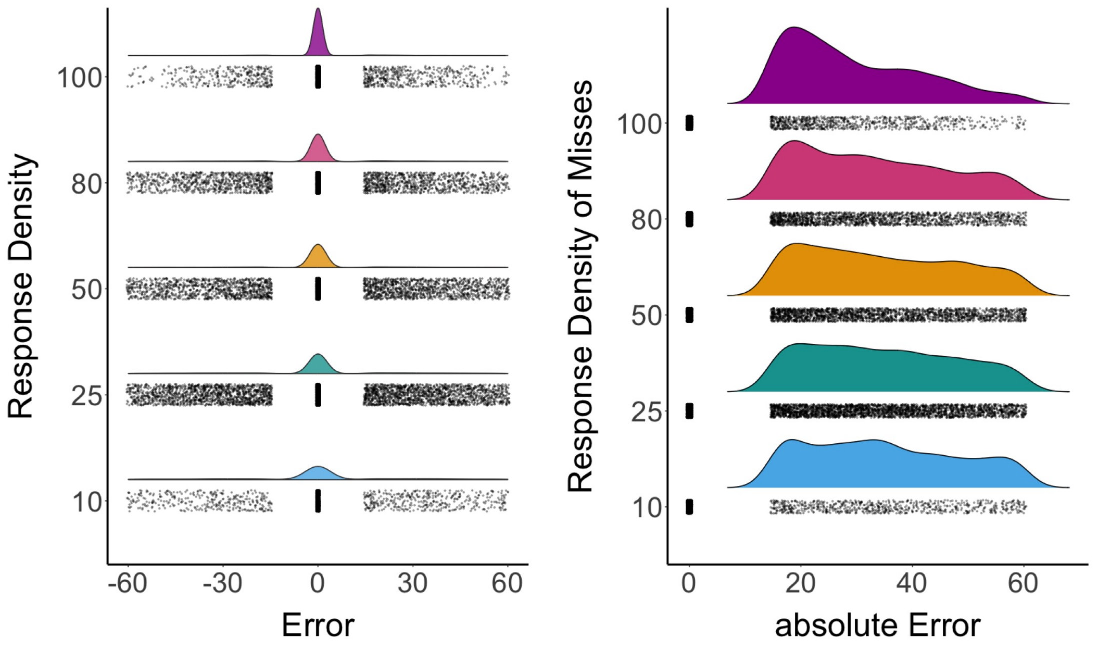

Figure S1. *2AFC response density showing non-continuity of responses.* **A**. Response density for All 2AFC responses in each probe probability condition. **B**. Responses density of Miss responses in absolute error. Miss responses were limited to be between 15-60 degrees.

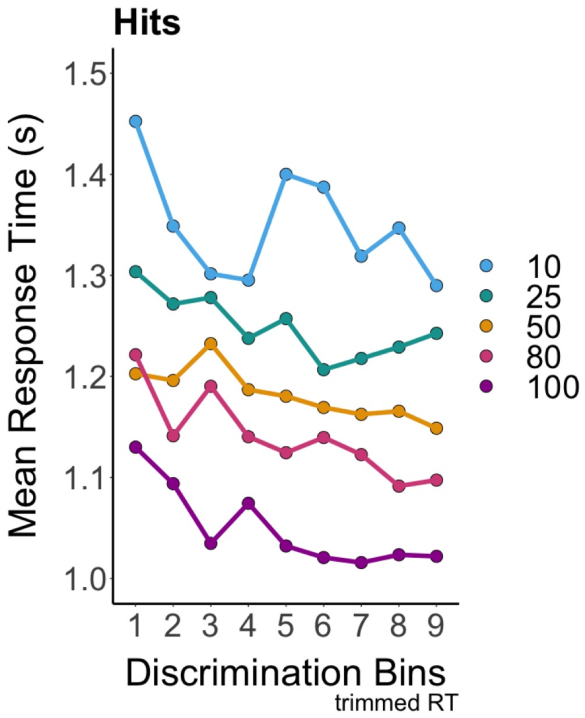

Figure S2*. Response time of 2AFC correct responses (hits) by decision difficulty (discrimination bin) for each attention allocation condition (probe probability condition)*.
